# Neuromyths in Music Education: Prevalence and Predictors of Misconceptions among Teachers and Students

**DOI:** 10.3389/fpsyg.2017.00629

**Published:** 2017-04-24

**Authors:** Nina Düvel, Anna Wolf, Reinhard Kopiez

**Affiliations:** Hanover Music Lab, Hanover University of Music, Drama and MediaHanover, Germany

**Keywords:** educational neuroscience, music students, music teachers, neuromyths, neuroscience of music, education

## Abstract

In the last decade, educational neuroscience has become increasingly important in the context of instruction, and its applications have been transformed into new teaching methods. Although teachers are interested in educational neuroscience, communication between scientists and teachers is not always straightforward. Thus, misunderstandings of neuroscientific research results can evolve into so-called *neuromyths*. The aim of the present study was to investigate the prevalence of such music-related neuromyths among music teachers and music students. Based on an extensive literature research, 26 theses were compiled and subsequently evaluated by four experts. Fourteen theses were selected, of which seven were designated as scientifically substantiated and seven as scientifically unsubstantiated (hereafter labeled as “neuromyths”). One group of adult music teachers (*n* = 91) and one group of music education students (*n* = 125) evaluated the theses (forced-choice discrimination task) in two separate online surveys. Additionally, in both surveys person-characteristic variables were gathered to determine possible predictors for the discrimination performance. As a result, identification rates of the seven scientifically substantiated theses were similar for teachers (76%) and students (78%). Teachers and students correctly rejected 60 and 59%, respectively, of the seven neuromyths as scientifically unsubstantiated statements. Sensitivity analysis by signal detection theory revealed a discrimination performance of *d'* = 1.25 (*SD* = 1.12) for the group of teachers and *d'* = 1.48 (*SD* = 1.22) for the students. Both groups showed a general tendency to evaluate the theses as scientifically substantiated (teachers: *c* = −0.35, students: *c* = −0.41). Specifically, buzz words such as “brain hemisphere” or “cognitive enhancement” were often classified as correct. For the group of teachers, the best predictor of discrimination performance was having *read* a large number of *media* about educational neuroscience and related topics (*R*^2^ = 0.06). For the group of students, the best predictors for discrimination performance were a high number of *read media* and the hitherto completed *number of semesters* (*R*^2^ = 0.14). Our findings make clear that both teachers and students are far from being experts on topics related to educational neuroscience in music and would therefore benefit from current education-related research in psychology and neuroscience.

## Introduction

Educational neuroscience, a comparatively new field, deals with the application of neuroscientific knowledge in educational practice (Alexander et al., 2012). Neuroscience has come to wide attention since the 1990s, due to new research made possible by a higher availability of neuro-imaging methods, such as fMRI. These new approaches offer innovative insights into the brain and its functions during cognitive activities such as reading, writing, calculating, and making music (for example Bangert et al., 2006; Kokal et al., 2011). Although many teachers are interested in the application of neuroscientific findings in the classroom, this is not always easy. Communication between scientists and teachers often follows the principle of *Chinese whispers*. Neuroscientists investigate complex structures and systems and, due to this intricacy, they are “not necessarily gifted at communicating with society at large” (Goswami, 2006, p. 6). Additionally, teachers are not always able and willing to become acquainted with the complex findings of science, and the “accurate transfer of research findings to the classroom is often difficult” (Dekker et al., 2012, p. 1). Misconceptions can easily occur at any of the mentioned stages of communication. Such misconceptions have been labeled as *neuromyths* (Goswami, 2006) and are generated by the misinterpreting, simplifying, misunderstanding and, in some cases, deliberate warping of neuroscientific research results (Organisation for Economic Co-Operation and Development, 2002, pp. 70–71). In this study, neuromyths are defined as scientifically unsubstantiated claims, which occasionally contain alarming amounts of misinformation (Goswami, 2006). According to Lilienfeld et al. (2010), there are several underlying mechanisms which facilitate the genesis and distribution of such myths. For instance, people desire simple answers and therefore easily believe in plausible proposals for solutions. People also notice and memorize information that supports their own assumptions more often than contradicting information. Finally, they often mistake causation for correlation (Lilienfeld et al., 2010, pp. 9–19).

The existence of neuromyths has been criticized many times (Organisation for Economic Co-Operation and Development, 2002; Goswami, 2006; Lilienfeld et al., 2010, p. 2), but in contrast to the great enthusiasm for educational neuroscience in the general public, the prevalence of neuromyths has rarely been investigated (e. g., Dekker et al., 2012). The prevalence of neuromyths among teachers remains a problem, as application in the classroom might not be beneficial or could even be harmful to learning processes. The so-called brain-based educational approaches can waste money, time, and effort (Goswami, 2006; Lilienfeld et al., 2010, pp. 8–9).

In a previous study, Dekker et al. (2012) investigated the prevalence and predictors of general neuromyths among teachers in different regions of the United Kingdom and the Netherlands. In their study, 242 primary and secondary teachers were asked to evaluate neuro-educational theses as *incorrect, correct*, or *do not know* in an online survey. Teachers wrongly believed in 49% of the neuromyths and correctly identified 70% of the true statements.

The present study used the same method as Dekker et al. (2012) with the difference that only neuro-educational theses about music were considered. Presumably, the field of music education is particularly sensitive to neuromyths. For example, questionable claims such as the *Mozart effect* (Rauscher et al., 1993; Chabris, 1999; Gruhn, 2008; Pietschnig et al., 2010) are widely distributed and believed. The original research by Rauscher et al. (1993) claimed that listening to Mozart's piano music temporarily enhances spatial temporal performance. This finding has been picked up on by the popular press and transformed into the more general claim that listening to Mozart's music increases intelligence, which is untrue and basically makes this assumption a myth. Based on this myth, especially in the United States an immense market for Mozart-effect CDs or music toys targeted toward babies emerged to foster the intelligence of very young children. This tendency reached its peak in 1998, when the governor of the US state of Georgia decided to donate a Mozart CD to every newborn child to foster the development of intelligence. Later replication and a meta-analysis (Chabris, 1999; Schellenberg, 2006, 2016) revealed that the so-called Mozart effect was not a permanent effect, but could also be caused by short-term arousal, evoked by other auditory stimulation such as music and audiobooks. However, the Mozart effect is still anchored in the public opinion.

The main aim of this study was to identify the prevalence of music-related neuromyths among music teachers and music students. The evaluation was conducted through an online survey, and additional person-characteristic variables were collected to determine relevant predictors of a high discrimination performance. As found in the study by Dekker et al. (2012), it was expected that the participants would perform better in the identification of theses with scientific substantiation (% hits) compared to neuromyths (% correct rejections). However, due to the public admiration for musical child prodigies (McPherson, 2016) and the popularity of the Mozart effect (Lilienfeld et al., 2010, pp. 45–49), we assumed that the unquestioned prevalence of neuromyths among music teachers and students would be higher when compared to teachers of other subjects. As an extension of the original study by Dekker et al. (2012) which focused on primary and secondary school teachers, we considered two different populations to investigate cohort effects of discrimination performance: music teachers at public schools (Study 1) and music students enrolled in teacher education programs (Study 2). It was assumed that—due to greater temporal and local proximity to current academic discussions—the younger generation of students would be more sensitive to neuromyths and show a higher detection rate. In comparison, older teachers, while being very experienced in classroom teaching, are not necessarily involved in current academic discussions and might therefore only possess the scientific knowledge that was up-to-date when they graduated. Of course, as an exception to this rule, there might be teachers with decades of experience who are still well-informed on current findings. Finally, the present study investigated which neuromyths are most and least prevalent so that we can determine/identify knowledge gaps in subsections of educational neuroscience.

## Materials: selection of theses

In a first step, 26 neuroscientific music-related theses were identified and extracted from the literature: The authors of this study started with a close reading of popular scientific books about music psychology, neurosciences of music, and related topics. In this process, salient statements were extracted and condensed into single theses maintaining the original wording. Based on first evidence, some of these theses seemed to be scientifically substantiated, and some seemed to be neuromyths. The neuromyths were either mentioned in the literature (meaning the literature supported the wrong information) or the author knew about the myth, mentioned it directly or indirectly, and corrected the misconception by presenting scientifically based information.

In a second step, these 26 theses were evaluated by four experts from the fields of neuroscience of music or music education (with a background in neuroscience of music education). The four experts received their doctoral degrees in the years 1967, 1983, 1996, and 2011. Two out of the four experts are active professors at German universities; one is a retired scholar, and the fourth expert is a post-doc researcher at a university. The four experts represent the research fields of music education, systematic musicology, music physiology/musicians' medicine, and educational music psychology in Germany. Their Hirsch indices are 5, 5, 16, and 49 (source: Google Scholar). The experts evaluated the collection theses against the background of existing evidence (5-point scale: *clearly wrong, commonly wrong, no clear decision, commonly true*, or *clearly true*), relevance in the current music educational discourse (3-point scale: *high, medium, low*), and required expertise for evaluation (3-point scale: *a little, some, a lot*). The assessments of all 26 theses, including arguments for and against consideration in this study, are shown in Supplementary Table 1. The table also provides the original German wording and the English translations. For consideration in the online survey, the evaluation of the experts had to show an unambiguous picture and to be supported by reliable literature such as meta-analyses. Finally, 14 of the 26 theses (seven neuromyths and seven substantiated statements) were considered as clearly classifiable and were tested in the survey.

**Figure 1:**
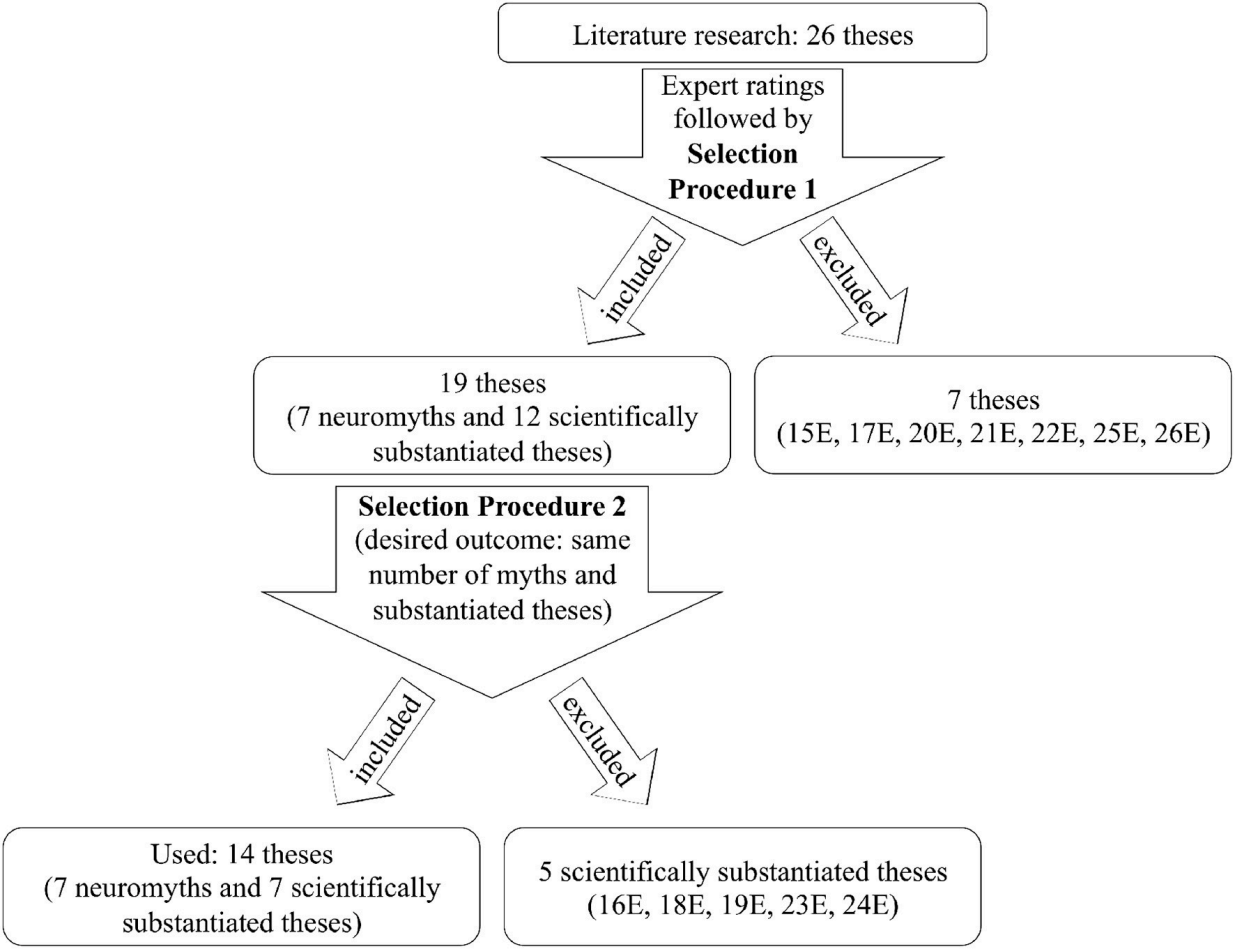
**Flowchart of the selection process of theses**.

The selection process for the final collection of 14 theses can be described in detail as follows (see Figure 1): After the expert ratings, the selection of theses for the two studies was discussed by the authors of this investigation. In a first step (“Selection Procedure 1,” see Figure 1), those seven theses were excluded for which no unanimous agreement could be reached as to whether they could be scientifically substantiated or should be regarded as neuromyths. For example, Thesis 15E (“Mozart effect”) is still being discussed in the scientific community and is seemingly related to 17E (“Improvement of spatial-visual skills by music listening”). As a common feature for the 19 theses that were included in the study, no thesis was assessed with more than one contrary evaluation by expert reviewers. The desired outcome of the second step (“Selection Procedure 2”) was an equal number of scientifically substantiated theses and neuromyths. As only seven neuromyths could be clearly identified among the 19 remaining theses, additionally five out of the 12 scientifically substantiated theses had to be excluded. This decision was guided by reasons such as the triviality of the statement (e.g., 24E “Training of motor skills through instrumental practice”), limited relevance for discourse in music education (e.g., 19E “Repeated listening increases liking for music”), or a high amount of expertise required for evaluation (e.g., 16E “Better memory performance in musicians,” 18E “Learning success and same tempo during learning and retrieval,” 23E “Joint singing facilitates social bonding”).

To control for the same complexity of the scientifically substantiated theses and the myths, the experts' ratings of the required expertise for evaluation were averaged. The mean difficulty (1 = low, 2 = medium, 3 = high) of myths ranged from 2.25 to 3.00 with an overall mean of *M* = 2.55. The mean difficulty of scientifically substantiated theses ranged from 2.00 to 3.00 with an overall mean of *M* = 2.54. Although the difficulty of evaluation for both groups of theses was above average, no difference between types of theses was found.

### Inter-rater agreement

The selection of theses was validated by the objective criterion of three intra-class correlation analyses (ICC). Inter-rater agreement of the expert raters was expected to be higher for selected theses compared to those theses not considered. Based on the ICC generalizable random model and an average agreement of raters (Shrout and Fleiss, 1979), the following intra-class correlations in the experts' ratings were found: for all 26 theses ICC(2,4) = 0.66; for the 14 selected theses ICC(2,4) = 0.77; and for the 12 non-selected theses ICC(2,4) = 0.43. Benchmarks for the quality of inter-rater agreement indicate that an ICC > 0.70 should be regarded as an indicator of “good” reliability (Wirtz and Caspar, 2002, p. 160), which was the case for those 14 theses considered for our investigation.

## Study 1

### Method

#### Procedure

In analogy to Dekker et al. (2012), the evaluation of the theses was conducted in an online survey (Reips, 2012). The link for invitation was distributed via several occupational associations of music teachers at public schools: the German National Association for Music Education (Bundesverband Musikunterricht), Association of German School Musicians (Verband deutscher Schulmusiker), and the German Society for Music Psychology (Deutsche Gesellschaft für Musikpsychologie). Additionally, the questionnaire was circulated via internet fora and social media.

As a cover story, the study appeared as a survey about neuroscientific knowledge and its application in music lessons in schools. To prevent arousing suspicion, the term “neuromyth” was never mentioned. The survey started with some sociodemographic questions and questions about the subject's interest in and previous experience with neuroscience and educational neuroscience. Subsequently, the participants were asked to evaluate the theses: In a 2-AFC paradigm, they had to decide whether the claims were “scientifically substantiated” or “scientifically unsubstantiated” (German terms: “wissenschaftlich belegt,” “wissenschaftlich nicht belegt”). The order of the theses was randomized, and the completion of the questionnaire took <15 min, which is in line with recommendations for online experiments (Reips, 2012).

#### A priori power analysis

For the a priori power analysis, Type I and Type II errors were set to α = 0.05 and β = 0.20, with an expected test power of 1-β = 0.80. The effect size (sensitivity *d'*) was assumed to be small (*d*' = 0.5). In compliance with the 2AFC-paradigm (two-alternative forced choice) within signal detection theory (Macmillan and Creelman, 2005), the necessary minimum sample size was *N* = 89 (Ennis and Jesionka, 2011, p. 380).

#### Ethical approval

The study was performed in accordance with relevant institutional and national guidelines and regulations (German Psychological Society, 2005) and with the principles expressed in the Declaration of Helsinki. Formal approval of the study by the Ethics Committee of the Hanover University of Music, Drama, and Media was not mandatory, as the study adhered to all the required regulations. Anonymity of participants and confidentiality of their data were ensured. They were informed about the objectives and the procedure of the survey as well as the option to withdraw from the study at any time without providing reasons or having any repercussions. All participants gave their informed consent online in accordance with the guidelines of the Hanover University of Music, Drama, and Media, by ticking a checkbox.

#### Participants

One hundred and thirty two teachers participated in the online survey. Of these, 38 did not complete the questionnaire, and three disagreed with the data storage for scientific purposes. The valid data of 91 participants from all over Germany remained to be analyzed. Most of the participants taught at various secondary schools (73.6%), while only some taught at primary schools (18.7%). Of the remaining teachers, 7.7% selected “other” as type of school. The schools from which participants were drawn can be considered a random selection of schools in Germany. The average age of the teachers was *M* = 43.0 years (*SD* = 10.3 years) ranging from 25 to 68 years (60.4% of the teachers were female).

#### Measurement of control and predictor variables

As a control variable, participants were asked to estimate their subjective belief, on a visual analog scale (1–101), in the relevance of genetic endowment compared to the relevance of environmental factors on learning success (*genes vs. environment*). Participants also indicated their highest academic qualifications: This information led to a variable indicating whether participants were studying toward a teaching career or not. Furthermore, we asked if they had received a PhD degree. Next, they indicated their interest in neuroscience and whether they considered this knowledge valuable for teaching practice (*application of neuroscience*; 4-point scale: *none at all, little, some, a lot*). They were asked whether they had participated in any further training in the field of brain research (*training in educational neuroscience*) and whether they had knowledge of educational approaches that claim to be neuroscience-based (*knowledge of teaching methods*). For further analyses, we calculated a sum score for *knowledge about neuroscience* (interest and application; “no” or “yes”) and *knowledge about educational neuroscience* (training and teaching methods; 4-point scale: *none* to *a lot*). From lists of books, journals, and websites about neuroscience or related topics, participants selected the media they had read. Additionally, there were the options “I have read another topic-related book/journal/website that is not listed” and “I haven't read a topic-related book/journal/media” for each type of media. The total number of read books, journals and websites was calculated (*number of read media*). See Table 1 for descriptive statistics of the predictor variables.

**Table 1:** **Descriptive data for teachers' characteristics (***n*** = 91 if not noted otherwise)**.

**Predictor variable**	**Descriptive statistics**			
	**Yes**		**No**	
Studied to become a teacher	73.6%		26.4%	
PhD degree (*n* = 89)	18.0%		82.0%	
	**Modus**		**Median**	
Knowledge about neuroscience (sum score)	8		7	
Knowledge about educational neuroscience (sum score)	3		3	
	***M***	***SD***	**Min**	**Max**
Genes vs. environment	56.21	19.67	2	91
Number of read media	2.47	2.29	0	9

*For details see text*.

### Results

**Figure 2:**
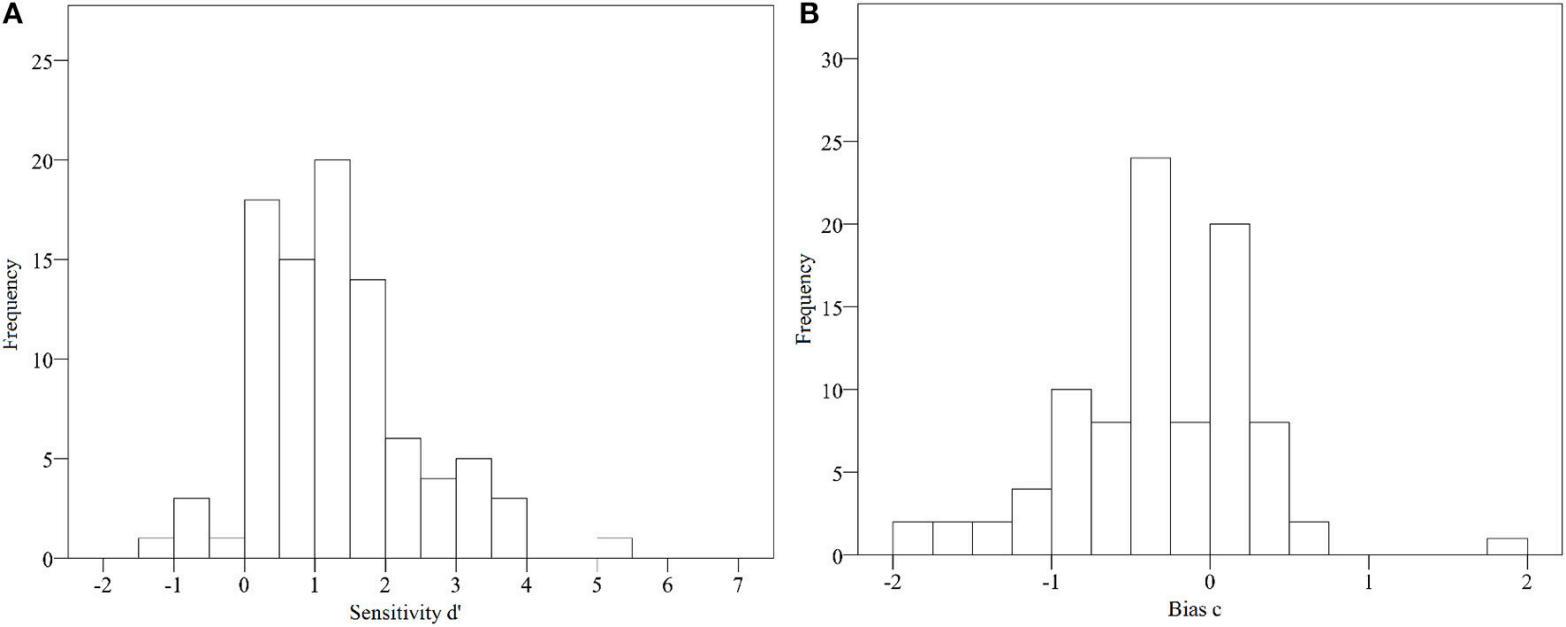
**(A,B)** Histogram of sensitivity *d'* (left) and response bias *c* (right) for the sample of music teachers (y-axis indicates frequencies).

The data were analyzed using a signal detection spreadsheet for Microsoft Excel 2010 (Sorkin, 1999) and the Statistical Package for the Social Sciences (SPSS V23). For all analyses, the threshold for the Type I error was set to α = 0.05. The evaluation of the selected 14 theses was analyzed according to Signal Detection Theory (Macmillan and Creelman, 2005). Indicators for discrimination performance (sensitivity *d*' and response bias *c*) were calculated (for distribution of discrimination performance values and response bias see Figures 2A,B). The average sensitivity among all teachers was *d*' = 1.25 (*SD* = 1.12, see Table 2 for details), which corresponds to a medium to large effect (Ennis and Jesionka, 2011, p. 380). The average response bias was *c* = −0.35 (*SD* = 0.58), which means that teachers showed an overall tendency to generally evaluate the theses as scientifically substantiated. Correlation coefficients between independent variables and sensitivity were calculated and are listed in Supplementary Table 2. The percentage of correct answers among neuromyths and among scientifically substantiated theses was calculated. On average, 76.1% of the seven scientifically substantiated theses were answered correctly, whereas only 59.5% of the neuromyths were correctly identified as such (see Table 2). The percentage of correct answers for every single thesis revealed the discrimination performance of participants on music educational sub-topics, as can be seen in Table 3.

**Table 2:** **Descriptive data for sensitivity, response bias and correct answers among music teachers and music students**.

	**Sensitivity *d' M* (*SD*)**	**Response bias *c M* (*SD*)**	**Correct answers to scientifically substantiated theses (%)**	**Correct answers to neuromyths (%)**
Teachers (*n* = 91)	1.25 (1.12)	−0.35 (0.58)	76.1	59.5
Students (*n* = 125)	1.48 (1.22)	−0.41 (0.71)	77.7	59.3

*Statistical tests for between-groups differences did not reach significance [d': t_(214)_ = −1.40, p = 0.16; c: t_(214)_ = 0.75, p = 0.45]*.

**Table 3:** **Correct answers among teachers and students for each of the seven neuromyths and seven scientifically substantiated theses (M = myth, S = scientifically substantiated)**.

**Thesis ID**		**Correct answers (%)**	
		**Teachers**	**Students**
**NEUROMYTHS**			
4M	Certain music genres require special ways of listening attitude. For classical music, only an intellectual listening style is appropriate.	96.7	90.4
1M	Excellent classical musicians are on average more intelligent than non-musical graduates of a university program.	75.8	80.8
3M	Those who listen passively to classical music during certain learning phases have advantages over those who do not listen passively to music.	68.1	64.8
2M	Music education improves one's performance in calculus significantly.	62.6	51.2
5M	Right-handers process speech in the left hemisphere of their brains and music in the right.	39.6	62.4
7M	The ability to improvise on the piano is controlled by the right hemisphere. Special exercises can enhance the performance of this hemisphere.	44.0	40.8
6M	Cognitive abilities, e.g., intelligence in children, can be effectively enhanced by music education.	29.7	24.8
**SCIENTIFICALLY SUBSTANTIATED THESES**			
8S	Musicians show a strong neurophysiological “coupling” between hearing and motor movement. This link was developed by intensive training.	79.1	87.2
12S	The anatomic structure of the brain changes through intensive practice of an instrument.	81.3	79.2
13S	Music education can enhance language skills.	82.4	76.0
11S	Musicians can process music faster, more precisely and more efficiently than non-musicians.	81.3	74.4
10S	Although not hearing-impaired, some people cannot understand tones, melodies and rhythms.	72.5	80.8
14S	The processing of auditory information is trained by music listening.	73.6	78.4
9S	The influence of passive listening to music during nonmusical activities depends, for example, on a person's degree of musical sophistication, the emotional effect and the character of the music.	62.6	68.0

To determine possible predictors for the discrimination performance, a multiple regression analysis was conducted (method: stepwise; criteria for entering and removing variables to the model: probability of *F* ≤ 0.05, *F* ≥ 0.10, respectively). The following predictor variables were entered: *age, gender, type of school* (primary school, secondary school, and other school types coded as dummy variables), *studied to become a teacher* (dummy variable for the highest qualification from secondary education was used), having a *PhD degree*, relevance of *genetic endowment and environmental factors* on learning success, *knowledge about neuroscience* (sum score of interest in and application of neuroscience), *knowledge about educational neuroscience* (sum score of training and teaching methods about educational neuroscience), and *number of read media*. Missing values were replaced by the mean.

As shown in Table 4, the analysis resulted in a regression model with the predictor variable *number of read media*. The explained variance accounted for 6.2% of the sensitivity score, which corresponds to a small effect size (Ellis, 2010, p. 41). The only predictor was the number of books, journals and websites read (β_*standardized*_ = 0.249).

**Table 4:** **Multiple regression predictors of discrimination performance (sensitivity d') for the sample of teachers**.

**Predictors**	***B (SE)***	**β*_*standardized*_***	***p***	**95% CI for** β	
				**Lower**	**Upper**
Intercept	0.951 (0.169)	–	0.000	0.616	1.287
Number of read media	0.121 (0.050)	0.249	0.017	0.022	0.221

*R^2^ = 0.062*.

In addition to the linear regression, and due to the small proportion of explained variance, we also applied a more robust statistical procedure: classification and regression trees as well as a random forest regression (Han et al., 2012) by means of the *party* package in the R Project for Statistical Computing (Strobl et al., 2009; Hothorn et al., 2017). We used the same eight predictor variables as for the multiple regression analysis to predict the sensitivity score of each participant. The threshold for the Type I error was set to α = 0.10. The results from the multiple regression analysis (Table 4) were replicated by the regression tree: Those students who read no media showed a lower discriminability for substantiated theses and myths (*M* = 1.21 for *n* = 77) than those who read one or more media (*M* = 1.91 for *n* = 48).

To find a more stable model than a single regression tree, we calculated a random forest regression and the permutation accuracy importance of the model's variables (Strobl et al., 2009). The permutation accuracy importance is a relative metric measure of variable importance. It gives information about the importance of variables in the random forest regression in such a manner that all values smaller than the absolute value of the most extreme negative importance are ignored. However, its value is not standardized and only allows relative conclusions about the variable importance within a particular random forest regression. The random forest regression also identified the *number of read media* as the most important predictor (permutation accuracy importance of 0.129) and *knowledge about neuroscience* as the second most important predictor (permutation accuracy importance of 0.039). The latter was the only additional predictor with a higher importance than the absolute value of the next largest negative predictor (permutation accuracy importance of |−0.022|). This random forest regression model only accounts for 2.4% of the variance in the sensitivity score.

### Discussion

The average sensitivity score shows a medium effect of being able to discriminate between substantiated and unsubstantiated music-related neuro-educational theses among school teachers. Yet, considering that teachers ought to be experts on topics related to education and learning, a better ability to discriminate between true and false scientific statements would have been desirable. The negative *response bias* indicates a tendency to evaluate theses on neuro-educational topics as scientifically substantiated. This general tendency to generally believe in explanations with neuroscientific content has also been shown in other studies (e.g., Weisberg et al., 2008). Since our study was presented as a study about neuro-educational knowledge (no mentioning of “neuromyths”), no suspicion was raised among the participants.

The *percentage of correct answers relating to neuromyths* and *relating to scientifically substantiated theses* reveals that teachers are more uncertain in evaluating neuromyths than in evaluating scientifically substantiated theses, as was hypothesized. The percentage of correct answers for every single thesis (see Table 3) reveals topics that are especially prone to misjudgment. For example, most of the teachers were convinced that music lessons are an effective way to enhance the cognitive abilities (e.g., intelligence) of children (Thesis 6M). Although this issue has been clarified by several publications in the past on the national and international level (e.g., Federal Ministry of Education Research (Ed.), 2006; Schellenberg, 2016), convictions based on popular neuro-educational myths are still widely spread. Popular media often support these music-related neuromyths by oversimplification. The much discussed Mozart effect is only one well-known example, although it has been scientifically debunked (Pietschnig et al., 2010). Further deficits become apparent in theses on the lateralization of brain functions: Many of the teachers were convinced that music is primarily processed in the right hemisphere of the brain and speech in the left one (Thesis 5M). The same naïve view of hemisphere specialization is the case for the ability to improvise on the piano: Many participants were convinced that this skill is controlled by the right hemisphere (Thesis 7M). This shows that the actual view of interconnected modules for the processing of music (Altenmüller, 2001) has not received much attention by many teachers.

Although teachers were generally prone to believing in music-related neuromyths, they outperformed the teachers in the study of Dekker et al. (2012) by reaching a higher percentage of correct answers relating to scientifically substantiated theses and to neuromyths. The hypothesis that theses on music-related topics are even more believable than those not on musical topics could not be supported.

The multiple regression analysis shows that having *read* many *media* (books, journals, and websites) about educational neuroscience and related topics predicts a high discrimination performance (correlation with a small effect size of *r* = 0.25, see Supplementary Table 2). Further analyses of correlations (also see Supplementary Table 2) revealed a (non-significant, *p* = 0.19) link between age and sensitivity (*r* = −0.14), which indicates that younger teachers might be more capable of critical evaluation of neuro-educational theses as “scientifically substantiated” or “scientifically unsubstantiated.” One explanation of this effect might be that the increasing emphasis on educational neuroscience over the last decades may have paid off. This last finding leads to the question whether music students are more competent and show a higher discrimination power than do music teachers. This hypothesis was tested in Study 2, which was started 5 months after Study 1.

## Study 2

In Study 2 the same theses (see Table 3) were evaluated by music students from German Universities who were studying to become teachers at public schools. Additionally, it was hypothesized that students with a higher number of study semesters would achieve a higher sensitivity.

### Method

The method was nearly identical to Study 1; the differing aspects are mentioned in the following sections.

#### Procedure

The students' version of the online survey was sent via e-mail to teaching staff at German Universities who circulated the information about the online survey among their music students who were enrolled in teacher education programs to become a teacher. The questionnaire was also distributed via social media.

#### A priori power analysis

As in Study 1, *N* = 89 participants were required to guarantee a sufficient level of test power.

#### Participants

In total, *n* = 138 Students participated in the online survey, whereas *n* = 13 had to be excluded from the data analysis for several reasons: First, *n* = 10 did not complete the questionnaire; *n* = 2 were enrolled in other study programs not aiming at a teaching career; and one participant obviously selected options by a meaningless response pattern. Thus, Study 2 acquired a valid sample of *n* = 125 participants from all over Germany. Most of the participants were studying to teach at secondary schools (90.4%), while a few were preparing to teach at primary schools (9.6%). The universities from which the participants were drawn can be considered a random selection of universities in Germany. The students were on average *M* = 22.4 years old (*SD* = 2.6 years, range: 17–32 years), and 59.2% of the students were female (one student did not reveal his/her gender, see also Table 5).

**Table 5:** **Descriptive data for the students' characteristics (***n*** = 125 if not noted otherwise)**.

**Predictor variable**	**Descriptive statistics**			
	**Modus**		**Median**	
Knowledge about neuroscience (sum score)	8		7	
Knowledge about educational neuroscience (sum score)	3		3	
Entering the teaching profession	4		4	
	***M***	***SD***	***Min***	***Max***
Genes vs. environment (*n* = 123)	62.36	21.13	16	101
Number of read media	0.81	1.53	0	11
Duration of studies (semesters)	5.93	3.61	1	18
Duration until completion	5.11	3.16	0	12
Total duration of academic studies	11.08	1.97	6	21

#### Measurements of control and predictor variables

The following measurements were taken exactly as they were in Study 1: genes vs. environment, interest in and appliance of neuroscience, teaching methods based on educational neuroscience, and the number of read media. Analogous to the question about further training in educational neuroscience, students were asked whether they had participated in courses on educational neuroscience or related topics. For the variables knowledge about neuroscience and knowledge about educational neuroscience, a sum score was calculated as in Study 1. The number of read media was also calculated as in Study 1.

Students were asked how many semesters they had studied (*duration of studies*) and how many semesters they would presumably need to finish their degree (*duration until completion*). Subsequently, these variables were added up and resulted in the anticipated *total duration of studies* of the students. Furthermore, students were asked whether they actually wanted to enter the profession of a teaching at a public school (4-level scale: *certainly not, possibly, probably, certainly*).

### Results

**Figure 3:**
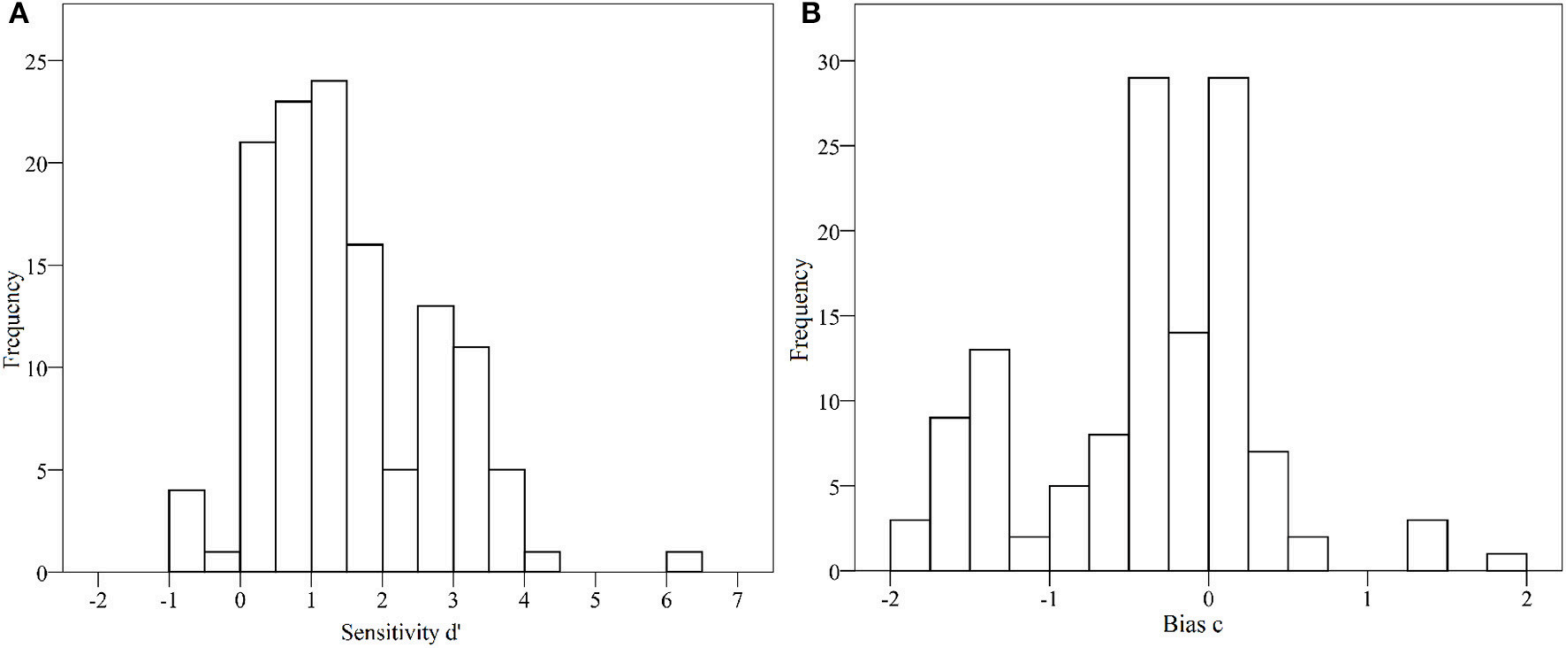
**(A,B)** Histogram of sensitivity *d'* (left) and response bias *c* (right) for the sample of music students (y-axis indicates frequencies).

The data were analyzed using the same software packages as in Study 1. The average sensitivity among all students was *d'* = 1.48 (*SD* = 1.22), which corresponds to a large effect size (Ennis and Jesionka, 2011, p. 380). The average response bias was *c* = −0.41 (*SD* = 0.71), which means that students showed an overall tendency to evaluate the theses as scientifically substantiated (see Figures 3A,B for distribution of *d'* values and response bias *c*), as did the teachers in Study 1. Correlations between predictor variables and sensitivity are shown in Supplementary Table 2. The calculation of the percentage of correct answers among neuromyths and among scientifically substantiated theses revealed that 77.7% of the 7 scientifically substantiated theses were on average answered correctly, whereas only 59.3% of the neuromyths were correctly identified as neuromyths (see Table 2).

The percentage of correct answers for every single thesis revealed the discrimination performance of participants on music educational sub-topics as reported in Table 3. Results for this group show a similar picture as do those for the teachers: The majority of students (75.2%) thought the neuromyth about music lessons and cognitive abilities (Thesis 6M) was true. Both neuromyths about lateralization (Theses 5M and 7M) also received many incorrect responses (37.6 and 59.2%, respectively, evaluated the neuromyths as being scientifically substantiated). Furthermore, the myth that music lessons improve performance in calculus (Thesis 2M) was also often believed to be correct: 48.8% of the students thought this had been scientifically substantiated.

In the next step, multiple regression analysis was conducted as in Study 1 (method: stepwise; criteria for entering and removing variables to the model: probability of *F* ≤ 0.05, *F* ≥ 0.10, respectively). The predictor variables were *age, gender, type of school* (primary school, secondary school, and other as dummy variables), relevance of *genetic endowment and environmental factors* on learning success, *knowledge about neuroscience* (sum score of interest in and application of neuroscience), *knowledge about educational neuroscience* (sum score of training and teaching methods in educational neuroscience), *number of read media, duration of studies, duration until completion, total duration of academic studies*, and *entering the teaching profession*.

The multiple regression in Table 6 shows that 14.1% of the variance of the sensitivity was accounted for by the predictors *read media* and *duration of studies* (*R*^2^ = 0.14 corresponding to a medium effect size; see Ellis, 2010, p. 41). Herein both predictors have approximately the same weight: for *number of read media* β_*standardized*_ = 0.237, and β_*standardized*_ = 0.240 for the *duration of studies*. For the sample of the teachers, the results from the multiple regression analysis (Table 4) could not be replicated by the regression tree: No significant predictor for this sample was found. By contrast, in the random forest regression only six out of the eight variables were selected for each calculated tree. This allowed for the appearance of collinear or otherwise interwoven variables. We found the *number of read media* to be the most important predictor (permutation accuracy importance of 0.119) and *knowledge about neuroscience* as another important predictor (permutation accuracy importance of 0.013), which was the only other predictor with a higher importance than the absolute value of the next largest negative predictor (permutation accuracy importance of |−0.011|). However, this model only accounts for 1.9% of the variance in the sensitivity score (*d*').

**Table 6:** **Multiple regression predictors of discrimination performance (sensitivity d') for the sample of students**.

	***B (SE)***	**β*_*standardized*_***	***p***	**95% CI for** β	
				**Lower**	**Upper**
Intercept	0.883 (0.202)	–	0.000	0.482	1.283
Number of read media	0.189 (0.069)	0.237	0.007	0.052	0.326
Duration of studies (semesters)	0.081 (0.029)	0.240	0.006	0.023	0.139

*R^2^ = 0.141*.

### Discussion

The average sensitivity revealed a large effect of being able to discriminate between substantiated and unsubstantiated music-related neuro-educational theses among students. Students also showed an overall tendency to generally consider the theses as scientifically substantiated. The percentage of correct answers related to neuromyths and to scientifically substantiated theses revealed that students were also more often off the mark in evaluating neuromyths than in evaluating scientifically substantiated theses. The relatively small percentage of correct rejections of neuromyths suggests that students are far from being experts in the fields of educational neuroscience.

Regarding the single theses, three neuromyths were correctly rejected by a surprisingly low percentage of participants: Many participants regarded theses which contained the expression “brain hemispheres” (Theses 5M and 7M), or claims that music education enhances cognitive abilities (Thesis 6M), as being true. The multiple regression analysis revealed that the number of *read media* and the hitherto completed *number of semesters* are the only relevant predictors for a high sensitivity. Both variables also show a significant correlation with the discrimination sensitivity (see Supplementary Table 2). Both *number of read media* and *number of semesters* can be considered as aspects of good education. Although we had assumed it would be a significant predictor, the age of the students was not relevant.

## General results

In the following section, the samples of teachers (Study 1) and students (Study 2) are compared concerning several predictor and outcome variables. Through comparison, a more extensive picture of the discrimination performance of music teachers and students enrolled in teacher education programs can be obtained. Furthermore, connections between discrimination performance in conjunction with teacher education nowadays and in preceding decades might be revealed. The discrimination performance and response bias for both groups are shown in Table 2. Although students showed a higher sensitivity than teachers, differences between groups were not significant [*d'*: *t*_(214)_ = −1.40, *p* = 0.16; *c*: *t*_(214)_ = 0.75, *p* = 0.45]. In search of reliable predictors of discrimination performance, we conducted a multiple regression analysis for all *N* = 216 participants of both groups (method: stepwise; criteria for entering and removing variables to the model: probability of *F* ≤ 0.05, *F* ≥ 0.10, respectively). Discrimination performance was used as the outcome variable, and the following predictor variables were selected: *group* (students = 0, teachers = 1), *age, gender, type of school* (primary school, secondary school, and other as dummy variables), relevance of *genetic* vs. *environmental factors* for learning success, *knowledge about neuroscience* (sum score of interest in and application of neuroscience), *knowledge about educational neuroscience* (sum score of training and teaching methods about educational neuroscience), and *number of read media*. Missing values were imputed by the mean.

The multiple regression analysis (see Table 7) shows that 7.8% of the total variance was accounted for by the predictors *number of read media* and *group*. This corresponds to a small effect size (Ellis, 2010, p. 41). Contributions of single predictors were as follows: *number of read media* β_*standardized*_ = 0.286; *group* β_*standardized*_ = −0.210. Thus, the higher the discrimination performance, the more books, journals and websites had been read by the participants of our studies, and the more likely it was that these participants were music students.

**Table 7:** **Multiple regression predictors of discrimination performance (sensitivity d') for the groups of teachers and students**.

	***B (SE)***	**β*_*standardized*_***	***p***	**95% CI for** β	
				**Lower**	**Upper**
Intercept	1.346 (0.107)	–	0.000	1.134	1.557
Group (1 = teachers vs. 0 = students)	−0.501 (0.172)	−0.210	0.004	−0.839	−0.163
Number of read media	0.165 (0.041)	0.286	0.000	0.083	0.246

*R^2^ = 0.078*.

**Figure 4:**
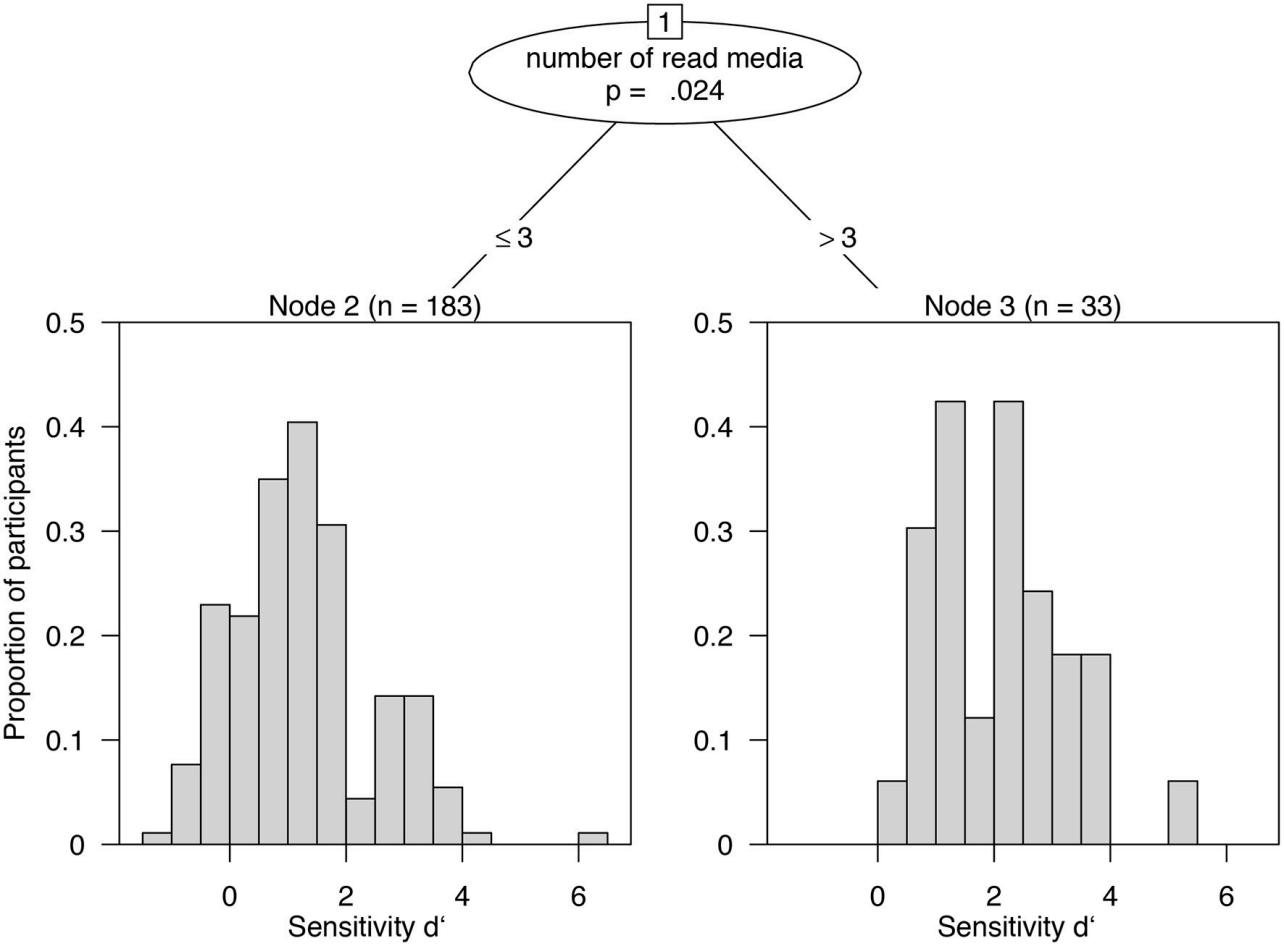
**Regression tree for sensitivity ***d'*** as outcome variable**. The only relevant predictor for high vs. low discrimination performance is the number of read media (≤3, >3).

In a final step, a classification and regression tree analysis (Witten et al., 2011) was conducted for the combined sample of teachers and students (see Figure 4). As a result, the only significant predictor in the regression tree was the *number of read media*. Those participants with a higher sensitivity (mean *d'* = 2.05) read more than three books, websites, and journals than the participants with a lower sensitivity (mean *d'* = 1.26) who only read three or fewer media.

Similarly, the additionally conducted random forest regression confirmed the same relevant predictor, *number of read media* (permutation accuracy importance of 0.076). The next important predictor, *knowledge about neuroscience*, only attained one-fifth of the value of the first predictor (permutation accuracy importance of 0.016) and showed only a slightly higher permutation accuracy importance than the absolute value of the largest negative predictor (permutation accuracy importance of |−0.014|). Therefore, all but the most relevant predictor *number of read media* and possibly *knowledge about neuroscience* should be neglected. This final model explains 19.8% of variance and supports the overall finding that domain-specific extensive reading activities correlate with a good discrimination performance.

## General discussion

This study investigated the prevalence and predictors of music-related neuroscientific myths among music teachers and music students. Accordingly, 14 theses (seven scientifically substantiated and seven neuromyths) were selected from the research literature on music and neuroscience and presented in an online survey in which music teachers and music students had to evaluate them as “scientifically substantiated” or “not scientifically substantiated.” Analyses revealed that many participants believe in neuromyths and that they are far from being experts on neuro-scientific topics. This was also reflected in the general tendency to their believing in the presented theses (and not to reject them as “false”). The predictors of a good discrimination performance, mainly the number of read media about psychology and neuroscience, can be associated with a good academic training. Thus, we assume that higher education in neuroscience and psychology, as well as a better general science education, is the key to prevent teachers of the next generations from believing in scientifically unsubstantiated neuromyths. As long as the quality of the read media, the tertiary education and the substance of the neuroscientific and neurodidactic knowledge is only quantified but not qualitatively evaluated, we can probably only expect small correlations from this noisy data for these and future studies. In other words, discrimination remains a difficult task, requires domain-specific training, and is not a self-evident fact.

In our study, participants showed a medium to large ability of discrimination. However, the difference between teachers and students did not reach statistical significance. Being a student (and not a teacher) was a relevant predictor for a higher sensitivity only in a multiple regression analysis (see Table 7); however, in a more robust analysis with regression trees and random forest regression, this result did not prevail. Therefore, our initial assumption that students with their current connections to the academic world would outperform teachers in discrimination could not be substantiated. Either the interest of current teachers in research results and their occupational training are higher than expected, or the scientific education of current music students is worse than expected.

The percentage of correct answers among scientifically substantiated theses and among neuromyths revealed that especially concerning the rejection of neuromyths, there is still much headroom for improvement in discrimination. Accordingly, the participants tended slightly to generally judge theses with neuroscientific jargon as scientifically substantiated (neuromyths 5M, 6M, and 7M). This is in line with the findings of Weisberg et al. (2008) who found that explanations containing neuroscientific words were more often trusted compared to similar explanations that did not use neuroscientific vocabulary. In our study, the three most-trusted neuromyths 5M, 6M, and 7M included neuroscientific terminology, such as “brain hemisphere” or “cognitive abilities,” while the neuromyths 1–4M contained no or less specialized neuroscience terminology. This explanation is, however, only a *post-hoc* finding and needs to be replicated with a more controlled approach in future studies with a special focus on music-related neuromyths.

Variables that were determined as relevant predictors for a good discrimination performance can all be associated, in general, with what is assumed to be good academic training associated with obtaining a high academic degree and the reading of relevant literature. Thus, a good education of students in subjects such as musicology, psychology, or neuroscience might result in a more critical attitude toward neuro-educational claims and lead to a better discrimination performance. Teachers should have the ability to critically evaluate neuro-educational theses, as the application of neuromyths in the classroom might waste the teacher's effort, learning time, and governmental funding. In the best case, the application of learning methods that are based on a myth does not support the learning process of the pupils. In the worst case, such learning methods might be more expensive and detrimental to the educational progress of the pupils. The critical handling of (pseudo-)scientific findings is necessary for a teacher to decide which methods can foster the learning process in music lessons and which do not. Additionally, aside from a possible delay in learning and the waste of taxes and resources, our technology- and health-centered culture has been promoted by scientific evidence over the last decades and centuries. Progress in society is best supported by finding out more about how the world works, and this has most successfully been done by a critical and ever-improving science.

Schools could use some of the available tools for the training of critical thinking, such as Sagan's (1997) “Baloney Detection Kit,” which helps people to “recognize a fallacious or fraudulent argument” (p. 210). Sagan's suggestions include applying well-known principles such as the “Occam's razor” principle or encouraging students to ask for “independent confirmation,” not to overly believe authorities, and to stay detached from hypotheses, which are “only a way station in the pursuit of knowledge” (p. 210) and need to be compared to other hypotheses.

By reconnecting these general tools for a scientific view of the world with, for instance, the widespread belief in the Mozart effect, schools and universities could promote critical and independent thinking first among their teachers and professors and then their students. Thus, both groups would improve their abilities to distinguish science from pseudo-science. The appeal of the Mozart effect for music lovers is that it makes a connection between the ingenious structure of the composition and the structure of the listener's mind: If people listen to “intelligent” music, their minds become intelligent as well. Nonetheless, this explanation is by far not the simplest among other accurate ones (see the Occam's razor principle), nor was there an independent confirmation for this finding (such as a replication study from another lab).

The present study shows evidence that there is a gap between the state of research in neuroscience related to music education and the knowledge of current and future music teachers about these findings and neuromyths. The high prevalence of neuromyths accepted for being true might indicate a lack of communication between scientists and music educators. Presumably, both disciplines must approach each other in order to deal with this problem. The results of the multiple regression and random forest regression analyses suggest that good scientific training (as indicated by number of read media, knowledge about neuroscience and, for the students' sample, duration of studies) might be the key to a critical understanding of scientific evidence and the ability to this from pseudo-scientific claims.

## Author contributions

Conceived and designed the experiments: ND, RK, AW. Performed the experiments: ND, RK, AW. Analyzed the data: ND, AW, RK. Wrote the paper: ND, AW, RK.

## Funding

This research received no specific grant from any funding agency in the public, commercial, or not-for-profit sectors.

### Conflict of interest statement

The authors declare that the research was conducted in the absence of any commercial or financial relationships that could be construed as a potential conflict of interest.
