# Supplementary material for: Neuromyths in Music Education: Prevalence and Predictors of Misconceptions among Teachers and Students
Source: Front Psychol. 2017 Apr 24;8:629. doi: 10.3389/fpsyg.2017.00629 (PMC5401909; doi:10.3389/fpsyg.2017.00629)
Supplement: Supplementary file 1 [file Table1.docx]

Supplementary Material

**Neuromyths in music education: Prevalence and predictors of misconceptions among teachers and students**

*Frontiers in Psychology*. doi: 10.3389/fpsyg.2017.00629

Nina Düvel, Anna Wolf, Reinhard Kopiez*

**Correspondence:**

Reinhard Kopiez: reinhard.kopiez@hmtm-hannover.de

## Supplementary Table 1

Table S1. List of all 26 music-related neuro-educational theses with evaluations by the four experts on a 5-point rating scale (index “M” = myth, “S” = scientifically substantiated, “E” = excluded; numbers indicate frequencies of expert evaluations).

| German (original) | English (translation) | Reference | Comments on evaluation results and selection criteria | Rating of scientific evidence | | | | | Relevance for music education-al discourse | | | Required expertise for evaluation | | |
| --- | --- | --- | --- | --- | --- | --- | --- | --- | --- | --- | --- | --- | --- | --- |
|  |  |  |  | Clearly wrong | Mostly wrong | No clear decision | Mostly true | Clearly true | High | Medium | Low | Low | Medium | High |
| 1M Hervorragende klassische Musiker sind durchschnittlich intelligenter als Nichtmusiker oder als die nicht-musikalische Bevölkerung mit Hochschulabschluss. | Excellent classical musicians are on average more intelligent than non-musical graduates of a university program. | Altenmüller (2006, p. 69); Jäncke (2008, p. 193); Schellenberg (2004, p. 511) | Statement is not substantiated: Literature is unanimous in rejecting this statement. | 1 | 2 |  | 1 |  | 2 | 1 | 1 | 1 |  | 3 |
| 2M Durch Musikausbildung werden die Leistungen im Rechnen merkbar verbessert. | Music education improves one’s performance in calculus significantly. | Federal Ministry of Education and Research (2006, p. 41); Jäncke (2008, p. 143); Roden et al. (2014, p. 545); Vaughn (2000) | Especially the meta-analysis from Vaughn makes clear that there is no support for the assumption of a *significant* improvement. | 2 |  | 1 | 1 |  | 1 | 1 | 2 |  | 2 | 2 |
| 3M Das passive Hören von klassischer Musik führt während bestimmter Lernphasen zu Leistungssteigerung. | Those who listen passively to classical music during certain learning phases have advantages over those who do not listen passively to music. | Jäncke (2008, p. 212) | Passive listening to pleasant music can help learners while studying, but classical music plays no particular role. The effect of background music depends more on preference, personality and many other factors; see also Thesis 9S. | 1 |  | 3 |  |  | 1 | 2 | 1 | 1 | 1 | 2 |
| 4M Bestimmte Genres/Stilistiken erzwingen einen bestimmten Hörzugang. Für klassische Musik kommt z.B. nur ein intellektueller Hörzugang in Frage. | Certain music genres require special ways of listening attitude. For classical music, only an intellectual listening style is appropriate. | Jäncke (2008, p. 255) | Jäncke makes clear that the concept of listening attitudes is much more variable than the way it is described in this thesis. Expert ratings clearly support this skeptical view. | 3 | 1 |  |  |  | 3 |  | 1 | 1 | 1 | 2 |
| 5M Bei Rechtshändern wird Sprache in der linken und Musik in der rechten Hirnhälfte verarbeitet. | Right-handers process speech in the left hemisphere of their brains and music in the right. | Jäncke (2008, p. 296) | In the past, this thesis was considered to be true, but was refuted by many studies (revieved by Jäncke). Expert ratings were unanimous in rejecting this statement; only one expert abstained from his evaluation. | 2 |  | 1 |  |  |  |  | 3 | 1 |  | 2 |
| 6M Musikunterricht ist ein Weg, die kognitiven Fähigkeiten, z.B. Intelligenz, eines Kindes effektiv zu fördern. | Cognitive abilities, e. g. intelligence in children, can be effectively enhanced by music education. | Degé (2015); Federal Ministry of Education and Research (2006, p. 48; 2009, p. 9); Schellenberg (2004) | Small effects on selected cognitive abilities could be substantiated, but music education is no *effective* way for cognitive enhancement. | 1 |  | 2 |  | 1 | 1 | 1 | 2 |  |  | 4 |
| 7M Improvisations-fähigkeit am Klavier wird besonders durch die rechte Hirnhälfte gesteuert. Spezielle Übungen können die Leistungsfähigkeit dieser Hemisphäre steigern. | The ability to improvise on the piano is controlled by the right hemisphere. Special exercises can enhance the performance of this hemisphere. | Wiedemann (1985, p. 129) | Regarding Thesis 5M, there is no empirical proof for this strict lateralization claim. Expert ratings were unanimous in rejecting this statement; one expert abstained from his judgement | 1 | 1 | 1 |  |  | 1 | 2 |  |  |  | 3 |
| 8S Musiker besitzen eine starke neurophysiologische „Kopplung“ zwischen Hören und Spielbewegung. Diese haben sich die Musiker durch intensives Lernen aufgebaut. | Musicians show a strong neurophysiological “coupling” between hearing and motor movement. This link was developed by intensive training. | Jäncke (2008, pp. 110–111) | Literature research is unanimous in accepting this statement. |  | 1 |  | 1 | 2 | 2 | 1 | 1 |  | 2 | 2 |
| 9S Der Einfluss von passivem Musikhören hängt z. B. von der Musikbildung, der emotionalen Wirkung und den Eigenschaften der Musik ab. | The influence of passive listening to music during nonmusical activities depends, for example, on a person’s degree of musical sophistication, the emotional effect and the character of the music. | Jäncke (2008, p. 218); Kämpfe, Sedlmeier & Renkewitz (2011); Kopiez (2008, pp. 531-533) | Both literature research and expert ratings are unanimous in accepting this statement.. |  |  | 2 | 1 | 1 | 2 | 1 | 1 |  | 2 | 2 |
| 10S Es gibt Personen, die Töne, Melodien und Rhythmen nicht verstehen können, obwohl ihre Hörleistung nicht beeinträchtigt ist. | Although not hearing-impaired, some people cannot understand tones, melodies and rhythms. | Jäncke (2008, pp. 133–135) | This thesis refers to congenital amusia. Literature research and expert ratings are unanimous in accepting this statement. |  |  | 2 | 1 | 1 | 1 | 2 | 1 |  | 1 | 3 |
| 11S Musiker können Musik besser (schneller, genauer, effizienter) wahrnehmen und verarbeiten, als Nichtmusiker. | Musicians can process music faster, more precisely and more efficiently than non-musicians. | Jäncke (2008, p. 158) | Both literature research and expert ratings are unanimous in accepting this statement. |  |  |  | 4 |  |  | 1 | 3 | 1 | 2 | 1 |
| 12S Durch intensives Üben eines Instruments verändert sich die anatomische Struktur des Gehirns. | The anatomic structure of the brain changes through intensive practice of an instrument. | Federal Ministry of Education and Research (2006, p. 55); Hyde et al. (2009); Jäncke (2008, p. 336) | This thesis refers to the plasticity of the brain. Literature research and expert ratings are unanimous in accepting this statement. |  |  |  | 3 | 1 | 1 | 1 | 2 |  |  | 4 |
| 13S Durch Musikunterricht können sprachliche Fähigkeiten gefördert werden. | Music education can enhance language skills. | Degé (2015); Federal Ministry of Education and Research (2006, p. 31); Jäncke (2008, p. 389) | This claim is substantiated (especially for phonological awareness). Expert ratings were unanimous in accepting this statement. |  |  | 1 | 2 | 1 | 2 | 2 |  |  | 1 | 3 |
| 14S Durch Musik wird allgemein die Verarbeitung von auditorischen Informationen geschult. | The processing of auditory information is trained by music listening. | Roden et al. (2014, pp. 553–554); Roden, Grube, Bongard & Kreutz (2014, pp. 11–12); Roden, Kreutz & Bongard (2012, p. 6) | This thesis is closely linked to Thesis 11S; both literature research and expert ratings are unanimous in accepting this statement. |  |  |  | 3 | 1 | 2 | 2 |  | 1 | 1 | 2 |
| 15E Das zehnminütige Hören von Mozarts Musik begünstigt das anschließende Lösen räumlicher Aufgaben. | Listening to Mozarts’s music for 10 minutes a day facilitates the subsequent solving of spatial tasks. | Federal Ministry of Education and Research (2006, p. 11, 60); Hetland (2000, p. 136); Jäncke (2008, p. 23–58); Pietschnig, Voracek & Formann (2010, p. 322); Rauscher, Shaw & Ky (1993) | This refers to the so-called “Mozart-effect”, for which there are inconsistent findings in literature. Expert ratings were ambiguous. |  | 1 |  | 1 | 2 | 2 |  | 2 |  | 2 | 2 |
| 16E Musiker haben bessere Gedächtnisleistungen in verbalen Tests als Nichtmusiker. | Musicians show better memory performance in verbal tests than nonmusicians. | Federal Ministry of Education and Research (2006, p. 94); Jäncke (2008, pp. 96–105); Roden, Könen et al. (2014, p. 545) | This thesis was considered as scientifically substantiated, but we excluded it from the study as we needed an equal number of substantiated theses and myths. |  |  | 1 | 3 |  | 3 |  | 1 |  |  | 4 |
| 17E Durch Musik werden auch räumlich-visuelle Fähigkeiten, z. B. das räumliche Vorstellungsvermögen, trainiert. | Spatial-visual abilities, such as spatial imagination, are trained through music. | Federal Ministry of Education and Research (2006, p. 36); Hetland (2000, p. 220; Jäncke (2008, p. 117) | This was confirmed by Hetland (2000, p. 220), but expert ratings were ambiguous. . | 1 | 1 | 2 |  |  | 1 | 1 | 2 |  | 2 | 2 |
| 18E Die Lernleistung kann gesteigert werden, indem Musik mit gleichem Tempo beim Lernen und beim Abruf vorgespielt wird. | Learning success can be increased by listening to music with the same tempo during learning and retrieval. | Jäncke (2008, p. 227) | This was excluded due to ambiguous expert ratings. | 1 |  | 2 | 1 |  |  | 2 | 2 |  |  | 4 |
| 19E Das mehrfache Hören eines Musikstücks führt zu positiverer Bewertung. | Repeated listening to a song leads to more positive ratings. | Jäncke (2008, p. 247) | This is possibly a mere exposure effect; there is no evidence for scientific proof. |  |  | 2 |  | 2 |  | 4 |  |  | 2 | 2 |
| 20E Durch Musik kann eine beidseitige Aktivierung des Gehirns herbeigeführt werden, die wiederum zu einer besseren Lernleistung verhilft. | A two-sided activation of the brain can be induced by music, which leads to a better learning performance. | Jäncke (2008, p. 208) | Jäncke presents this thesis as a clear myth; expert ratings were ambiguous. | 1 |  |  | 1 | 1 |  | 3 |  |  | 2 | 1 |
| 21E Durch Musiktraining, besonders im Kindesalter, werden die beiden Gehirnhälften besser miteinander verknüpft. | Music training, especially in childhood, enhances the connection of the hemispheres. | Gaser & Schlaug (2003, p. 9243); Jäncke ( 2008, p. 340–342); Steele, Bailey, Zatorre & Penhune (2013) | This relates to the anatomical structure of the corpus callosum which links brain hemispheres. Early findings showing an increased corpus callosum strength in musicians could not be replicated by Gaser & Schlaug (2003). |  |  | 1 | 1 | 1 | 1 | 2 |  |  | 1 | 2 |
| 22E Durch Musikunterricht lassen sich bei Kindern soziale Kompetenzen fördern. | Social competencies in children can be fostered by music lessons. | Federal Ministry of Education and Research (2009, p. 66, 118 f.); Kirschner & Tomasello (2010, p. 361); Schellenberg (2004, p. 315; 2009, p. 114) | For this thesis there are controversial findings in literature and ambiguous expert ratings. |  | 1 | 1 | 2 |  | 2 | 1 | 1 |  | 2 | 2 |
| 23E Durch gemeinsames Singen wird die soziale Bindung gestärkt. | Singing together facilitates social bonding. | Kreutz (2014) | This is presumably correct in amateur choirs, but there is currently no broader data basis for a generalizable statement. |  |  | 2 | 2 |  | 1 | 3 |  |  | 2 | 2 |
| 24E Durch das Spielen eines Instruments wie  z. B. Klavier oder Geige werden die motorischen Fähigkeiten trainiert. | Playing an instrument such as the piano or the violin trains motor skills. | Jäncke (2008, p. 308f.); Roden, Könen et al. (2014, p. 545) | This was excluded due to the triviality of this finding. |  |  |  | 3 | 1 | 1 | 3 |  | 1 | 1 | 2 |
| 25E Der intensive Gebrauch digitaler Medien (z. B. Internet, Smartphones oder portabler Musik-wiedergabegeräte) kann die kognitive Leistungsfähigkeit von Kindern negativ beeinflussen. | The intense use of digital media (e.g., internet, smart phones or portable music players) can affect the cognitive performance of children negatively. | Spitzer (2012, pp. 221, 322) | For this thesis there are controversial findings in the literature research and ambiguous expert ratings. |  |  | 3 | 1 |  | 3 |  | 1 |  |  | 4 |
| 26E Die Nervenzellen im menschlichen Gehirn sind bereits bei der Geburt voll ausgebildet, im Laufe des Lebens sterben täglich welche ab. | The nerve cells in the human brain are fully developed at birth, and some deteriorate from day-to-day in the lifespan. | Spitzer (2012, p. 55) | For this there are ambiguous expert ratings. | 1 |  | 1 | 1 | 1 |  |  | 4 |  |  | 4 |

## Supplementary Table 2

Table S2. Correlations between the outcome variable *d’* (discrimination performance) and predictor variables for the groups of teachers and students

| Predictor variables | Teachers | Students |
| --- | --- | --- |
|  | Pearson correlation *r* | |
| Age | -.140^+^ | .140 |
| Genes vs environment | .101 | .092 |
| Number of read media | .249* | .294** |
| Duration of studies | - | .297** |
| Duration until completion | - | -.195* |
| Total duration | - | .213* |
|  | Spearman correlation *r_s_* | |
| Knowledge about neuroscience | -.081 | -.010 |
| Knowledge about educational neuroscience | -,164 | -.101 |
| Entering the teaching profession | - | .112 |
|  | Point-biserial correlation *r_pb_* | |
| Gender (0 = female, 1 = male) | .034 | .000 |
| Type of school (1 = secondary, 0 = other) | -.018 | .141 |
| Studied to become a teacher (1 = yes, 0 = no) | -,029 | - |
| PhD degree (1 = yes, 0 = no) | .233* | - |

*Note*. * *p* > .05 (two-tailed); ** *p* > .01 (two-tailed); ^+^ *p* = .186

References

Altenmüller, E. (2006). Neuronale Auswirkungen musikalischen Lernens im Kindes- und Jugendalter und Transfereffekte auf Intelligenzleistungen [Neuronal effects of musical learning in childhood and adolescence and transfer effects on intelligence performance]. In Bundesministerium für Bildung und Forschung (Ed.), *Macht Mozart schlau? Die Förderung kognitiver Kompetenzen durch Musik* [Does Mozart make one smart? Facilitation of cognitive abilities through music] (pp. 59–70). Bonn, Germany: Bundesministerium für Bildung und Forschung .

Degé, F. (2015). Die nächste Studie kommt bestimmt. Macht Musikunterricht intelligenter und sollten wir und das wirklich fragen? [The next study is definitely coming. Do music lessons make one intelligent and should we really ask that?] *Neue Musikzeitung, 64*(2), 28.

Federal Ministry of Education and Research (Ed.). (2006). *Macht Mozart schlau? Die Förderung kognitiver Kompetenzen durch Musi*k [Does Mozart make one smart? Facilitation of cognitive abilities through music]*.* Berlin, Germany: Federal Ministry of Education and Research.

Federal Ministry of Education and Research (Ed.). (2009). *Pauken mit Trompeten: Lassen sich Lernstrategien, Lernmotivation und soziale Kompetenzen durch Musikunterricht fördern?* [Cramming with trumpets: Can learning strategies, learning motivation and social skills be facilitated by music lessons?]*.* Berlin, Germany: Federal Ministry of Education and Research.

Gaser, C., & Schlaug, G. (2003). Brain structures differ between musicians and non-Musicians. *The Journal of Neuroscience: The Official Journal of the Society for Neuroscience, 23*(27), 9240–9245.

Hetland, L. (2000). Learning to make music enhances spatial reasoning. *Journal of Aesthetic Education, 34*(3/4), 179. doi:10.2307/3333643

Hyde, K. L., Lerch, J., Norton, A., Forgeard, M., Winner, E., Evans, A. C. et al. (2009). Musical training shapes structural brain development. *The Journal of Neuroscience: The Official Journal of the Society for Neuroscience, 29*(10), 3019–3025. doi:10.1523/JNEUROSCI.5118-08.2009

Jäncke, L. (2008). *Macht Musik schlau? Neue Erkenntnisse aus den Neurowissenschaften und der kognitiven Psychologie* [Does Mozart make one smart? New findings from neuroscience and cognitive psychology]. Bern, Switzerland: Huber.

Kämpfe, J., Sedlmeier, P., & Renkewitz, F. (2011). The impact of background music on adult listeners: A meta-analysis. *Psychology of Music, 39*(4), 424–448. doi:10.1177/0305735610376261

Kirschner, S. & Tomasello, M. (2010). Joint music making promotes prosocial behavior in 4-year-old children. *Evolution and Human Behavior, 31*(5), 354–364. doi:10.1016/j.evolhumbehav.2010.04.004

Kopiez, R. (2008). Wirkungen von Musik [Effects of music]. In H. Bruhn, R. Kopiez, & A. C. Lehmann (Eds.), *Musikpsychologie. Das neue Handbuch* [Music psychology. The new handbook] (pp. 525–547). Reinbek, Germany: Rowohlt.

Kreutz, G. (2014). Does singing facilitate social bonding? *Music & Medicine, 6*(2)*,* 51–60.

Pietschnig, J., Voracek, M., & Formann, A. K. (2010). Mozart effect–shmozart effect: A meta-analysis. *Intelligence, 38*(3), 314–323. doi:10.1016/j.intell.2010.03.001

Rauscher, F. H., Shaw, G. L., & Ky, K. N. (1993). Music and spatial task performance. *Nature, 365,* 611.

Roden, I., Grube, D., Bongard, S. & Kreutz, G. (2014). Does music training enhance working memory performance? Findings from a quasi-experimental longitudinal study. *Psychology of Music, 42*(2), 284–298. doi: 10.1177/0305735612471239

Roden, I., Könen, T., Bongard, S., Frankenberg, E., Friedrich, E. K. & Kreutz, G. (2014). Effects of music training on attention, processing speed and cognitive music abilities: findings from a longitudinal study. *Applied Cognitive Psychology, 28*(4), 545–557. doi: 10.1002/acp.3034

Roden, I., Kreutz, G. & Bongard, S. (2012). Effects of a school-based instrumental music program on verbal and visual memory in primary school children: a longitudinal study. *Frontiers in Psychology, 3,* 572. doi:10.3389/fpsyg.2012.00572

Schellenberg, E. G. (2004). Music lessons enhance IQ. *Psychological Science, 15*(8), 511–514. doi:10.1111/j.0956-7976.2004.00711.x

Schellenberg, E. G. (2009). Musikunterricht, geistige Fähigkeiten und Sozialkompetenzen: Schlussfolgerungen und Unklarheiten [Music lessons, mental abilities and social skills: Conclusions and uncertainties]. In Bundesministerium für Bildung und Forschung (Ed.), *Pauken mit Trompeten. Lassen sich Lernstrategien, Lernmotivation und soziale Kompetenzen durch Musikunterricht fördern?* [Cramming with trumpets: Can learning strategies, learning motivation and social skills be facilitated by music lessons?] (p. 114–124). Berlin, Germany: Federal Ministry of Education and Research.

Spitzer, M. (2012). *Digitale Demenz. Wie wir uns und unsere Kinder um den Verstand bringen* [Digital dementia: How we drive ourselves crazy and our children out of their minds]. München, Germany: Droemer.

Steele, C. J., Bailey, J. A., Zatorre, R. J. & Penhune, V. B. (2013). Early musical training and white-matter plasticity in the corpus callosum: Evidence for a sensitive period. *Journal of Neuroscience, 33*(3), 1282–1290. doi:10.1523/JNEUROSCI.3578-12.2013

Vaughn, K. (2000). Music and mathematics: Modest support for the oft-claimed relationship. *Journal of Aesthetic Education, 34*(3-4), 149–166. doi: 10.2307/3333641

Wiedemann, H. (1985). *Klavierspiel und das rechte Gehirn. Neue Erkenntnisse der Gehirnforschung als Grundlage einer Klavierdidaktik für erwachsene Anfänger* [Piano playing and the right hemisphere: New findings from brain research as a basis for piano didactics for adult beginners]. Regensburg, Germany: Bosse.
